# Poncet’s disease: two case reports

**DOI:** 10.1186/s13256-017-1260-0

**Published:** 2017-04-06

**Authors:** Fatima Adhi, Rabia Hasan, Mehreen Adhi, Syed Ali Hamid, Nousheen Iqbal, Javaid A. Khan

**Affiliations:** 1 Aga Khan University Karachi, Karachi, Pakistan; 2 Department of Medicine, Dow University of Health Sciences, Karachi, Pakistan; 3 Department of Medicine, Section of Pulmonary and Critical Care, Aga Khan University, Stadium Road, Karachi, Pakistan

**Keywords:** Tuberculosis, Tuberculous rheumatism, Poncet’s disease, polyarthritis

## Abstract

**Background:**

One of the rare presentations of active pulmonary or even extrapulmonary tuberculosis is polyarthropathy which is the involvement of multiple large and small joints in the body; a reactive constellation known as Poncet’s disease. This may sometimes be the sole manifestation of the disease before more obvious features develop. The pain experienced during polyarthritis can be crippling thereby limiting the mobility and activities of patients. Polyarthritis as a symptom of active tuberculosis can be easily misinterpreted for more common causes of polyarthritis such as rheumatological diseases that present similarly.

**Case presentation:**

We describe the case of a 25-year-old Asian woman and a 45-year-old Asian man who presented with active tuberculosis where polyarthralgia was the first and only symptom for many months followed by pulmonary and pleural manifestations. Both patients showed dramatic improvement with anti-tuberculous therapy. The total duration of therapy was 6 months.

**Conclusions:**

Based on our observations, we propose that tuberculosis be included among the differentials for patients with unusual presentation of joint pains, especially in endemic regions and/or susceptible populations.

## Background

Tuberculous rheumatism (Poncet’s disease) is a rare, reactive acute-onset polyarthritis associated with active extra-articular tuberculosis (TB) [1, 2]. It presents as symmetrical involvement of multiple joints without evidence of active infection and resolves without residual joint damage or long-term complications [2–4]. In clinical practice, the diagnosis is generally not considered unless the manifestations are not accounted for by any other disease process.

We present two patients with debilitating polyarthropathy who developed pulmonary and pleural TB later in the course of the disease. In the light of these observations we propose that TB be included in the differentials and work-up of polyarthritis, especially in endemic regions.

## Case presentation

### Case 1

A 25-year-old Asian woman presented with constant bilateral heel pain for 3 weeks with difficulty in walking. The pain significantly limited her routine activities. She also complained of migratory pain involving her ankles, knees, and hips of the same duration. The pain worsened with activity and there was no associated morning stiffness. She had never been sexually active. Her childhood immunizations were complete and included Bacillus Calmette–Guérin (BCG) vaccine at birth. There was no family history of TB, rheumatologic disease, or autoimmune disease.

On examination, there was mild swelling of her left ankle joint, without erythema, tenderness, or restriction of movement. All other joints, respiratory, and otorhinolaryngeal examinations were normal and she was afebrile. A complete blood count (CBC) was within normal limits. X-rays of the joints of her hands and knees were done which were normal without any evidence of erosion. She was prescribed non-steroidal anti-inflammatory drugs (NSAIDs), skeletal muscle relaxants, topical analgesics, and anti-histamines for symptomatic relief. However, a week later the pain worsened together with her symptoms compelling her to walk on tiptoe. There was severe bilateral heel pain and progression of joint involvement which now included her wrists and small joints of her hands bilaterally. She failed to respond to higher doses of NSAIDs and the addition of opioid analgesics to the regimen, and over the next few days she went on to develop low-grade fever of 37.6 to 37.8 °C (99.6 to 100 °F), accompanied by anorexia, fatigue, loss of 2 kg weight compared to her initial charting, and mild non-productive cough. Laboratory investigations revealed an erythrocyte sedimentation rate (ESR) of 72 mm/hour and C-reactive protein (CRP) of 4.9 mg/dl, which suggested an acute inflammatory etiology. Her level of creatinine phosphokinase (CPK) was normal. She was prescribed a tapering dose of steroids for 2 weeks, because of which there was an improvement in pain, but daily fever spikes of 37.8 °C (100.0 °F) continued. Her ESR decreased to 55 mm/hour and CRP to 1.9 mg/dl.

Ten days after her steroids were discontinued, she presented with extreme worsening of all her symptoms, a constant fever of 37.8 °C (100 °F), an additional 2 kg weight loss, and non-productive cough. Her CBC, serum electrolytes, and liver functions remained normal. Her ESR and CRP shot up to 70 mm/hour and 5.3 mg/dl respectively, while ferritin was 204 mg/dl. Rheumatologic investigations revealed positive anti-nuclear antibody (ANA) but anti-smooth muscle antibody (ASMA), anti-mitochondrial antibody (AMA), anti-double stranded deoxyribonucleic acid antibody (anti-dsDNA), and rheumatoid factor (RF) were negative.

**Fig. 1:**
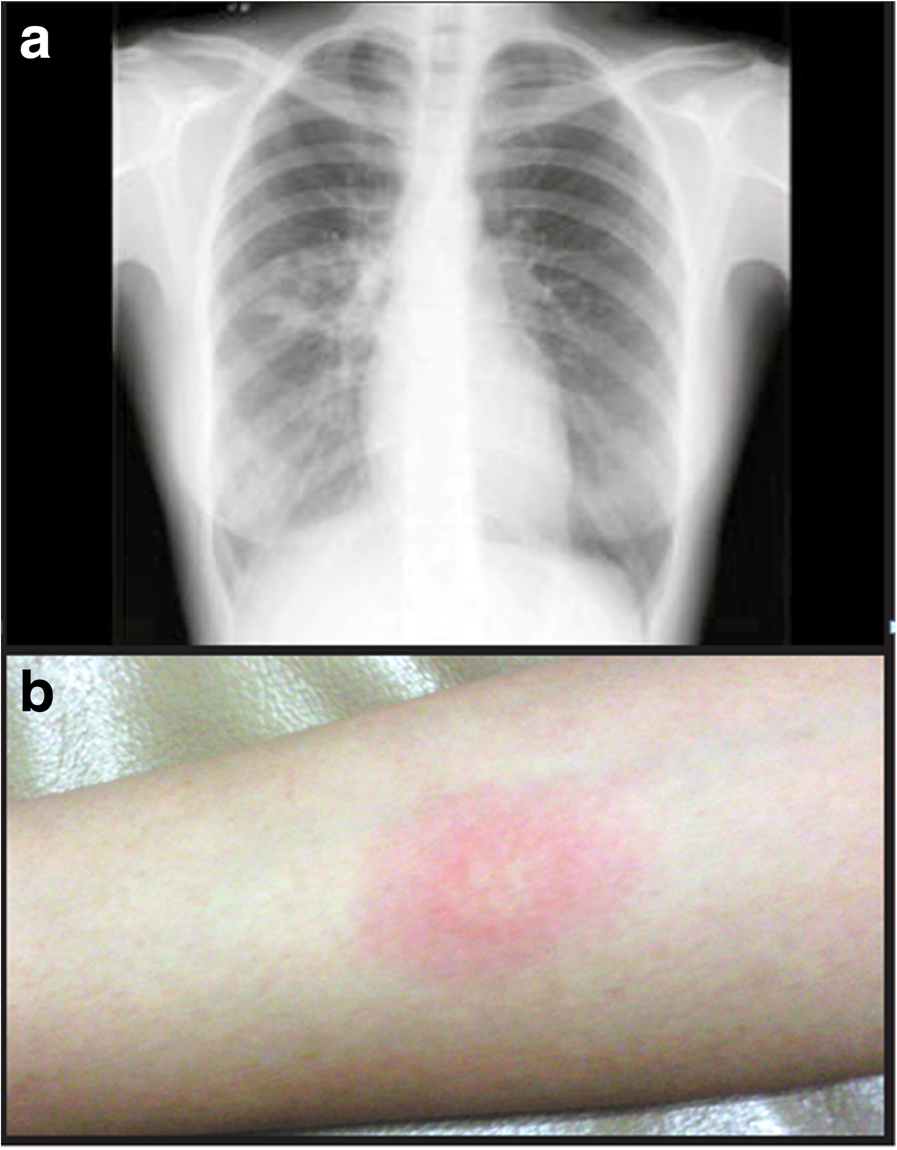
**a** Chest X-ray of the patient showing patchy perihilar infiltrates. **b** Purified protein derivate skin test showing induration of 22 mm, 72 hours after intradermal injection

A chest X-ray revealed patchy infiltrates in right perihilar region and lower lobe of her right lung without evidence of hilar lymphadenopathy or pleural effusion (Fig. 1a). A purified protein derivative (PPD) skin test resulted in induration of 22 mm at 72 hours (Fig. 1b). Previous records showed a negative PPD skin test 2 years ago, confirming recent seroconversion. Acid-fast bacilli (AFB) smears could not be obtained, since she had no sputum production, even with repeated saline nebulizations. Bronchoalveolar lavage was planned to confirm diagnosis, and synovial tissue cultures and joint aspirates from symptomatic joints were considered to rule out osteoarticular TB. However, she refused to offer consent for any invasive procedure.

Hence, with the provisional diagnosis of reactive polyarthritis associated with extra-articular TB, she started on standard anti-tuberculous therapy (ATT) with four-drug regimen (isoniazid, rifampicin, ethambutol, and pyrazinamide) and pyridoxine supplementation. There was alleviation of all the symptoms with no joint swelling and tenderness on examination; she became afebrile and her CRP normalized within a few days of starting therapy. Her ESR reduced to 33 mg/dl and returned to normal over the next 3 months. She continued on the four-drug regimen for 2 months and then she was treated with a two-drug regimen with isoniazid and rifampicin for 2 months. Three years following the completion of treatment, she remained free of symptoms, laboratory parameters were normal, and a chest X-ray showed resolution of infiltrative changes.

### Case 2

A 45-year-old Asian man presented with a 12-month history of pain in multiple joints without any prior trauma, and without accompanying swelling or erythema. He reported a considerable decline in his functionality with an inability to carry out even daily routine activities, being restricted to a wheelchair for the last few days. Of insidious onset, the pain was waxing, waning, and migratory in nature. It involved his ankles and knees bilaterally, and multiple small joints of his hands and spine. There was associated low-grade fever of ≤37.2 °C (≤99 °F) and 10 to 15 minutes of morning stiffness. He was being treated elsewhere with a variety of NSAIDs, steroids, and the disease-modifying anti-rheumatic drugs methotrexate and Salazopyrin (sulfasalazine) for provisional diagnoses of malaria, typhoid, and rheumatoid arthritis (RA) before presenting to us. These drugs gave mild relief from pain, but he continued to have fever.

On presentation he had a fever of 39.4 °C (103 °F), generalized lethargy, and a history of 23 kg loss of weight over 5 months. In addition, he complained of dyspnea for 2 weeks associated with cough productive of minimal amount of mucoid sputum. He had been vaccinated with BCG at birth. There was no family history of TB, rheumatologic disease, or autoimmune disease or any history of contact with anybody with active TB or similar symptoms. He was in a monogamous relationship. A physical examination revealed an obese man with no signs of inflammation or effusion in any joint. Breath sounds were decreased in the lung bases bilaterally accompanied with right supraclavicular lymphadenopathy.

**Fig. 2:**
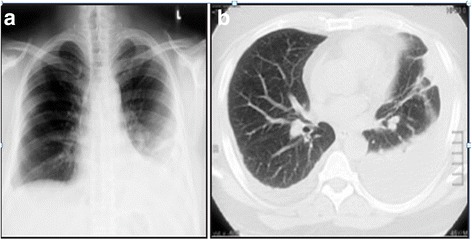
**a** Chest X-ray and **b** computed tomography scan of the chest showing bilateral pleural effusions, more significant on the left side

**Fig. 3:**
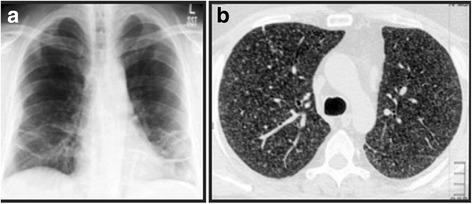
**a** Chest X-ray and **b** computed tomography scan of the chest showing multiple fine nodularities bilaterally in all lung fields

A chest X-ray revealed mild bilateral pleural effusion and left-sided lower lobe consolidation (Fig. 2a). A computed tomography (CT) scan of his chest re-demonstrated the effusions and also showed hilar lymphadenopathy (Fig. 2b). Aspiration revealed exudative effusion (protein of 5400 mg/dL, lactate dehydrogenase of 3274 IU/L) with lymphocytic pleocytosis (leukocyte count of 800 with 90% lymphocytes) and low glucose (51 mg/dL). A pleural fluid AFB smear and culture were negative. His hemoglobin level was 9.4 g/dL, ESR 111 mm/hour, and CRP 21.6 mg/dl. ANA (homogenous) and RF were all positive and anti-citrullinated cytoplasmic peptide antibody was 119. An X-ray of his joints did not reveal any joint erosions or abnormalities. A high-resolution CT scan of his chest and a repeated chest X-ray 1 week later revealed multiple fine nodules bilaterally in all lung fields consistent with miliary TB (Fig. 3).

He was subsequently started on standard ATT regimen (as for Case 1) and prednisone was tapered over the ensuing few months. He reported significant improvement of symptoms within 10 days of initiation of ATT. A chest X-ray 1 month later was normal with resolution of both effusion and nodularities. Over the next year, he improved further and maintained good health 5 months after completion of ATT.

## Discussion

In our patients we could not perform joint aspiration but the constellation of symptoms occurring in the presence of evidence of active tubercular lesions in their lungs without evidence of any joint erosion and abnormalities, pointed toward reactive tuberculous arthritis. In addition, the rapid resolution of symptoms following ATT confirmed the clinical suspicion.

The rarity of this presentation [5, 6], even in areas like Pakistan where TB is endemic, and the ambiguity surrounding its pathogenesis have allowed the controversy regarding its very existence to persist. In consequence, clinicians widely remain unaware of this presentation, possibly leading to under-diagnosis, mistreatment, and lack of concrete literature with only a few cases published over the years [7]. By definition, the arthritis is sterile [8], and bacterial involvement should first be ruled out. The dearth of data, however, has hindered the development of specific diagnostic criteria with most treatment being presumptive based on strong clinical suspicion and supporting radiological or laboratory evidence of active TB [7]. Osteoarticular TB should ideally be ruled out by identification of mycobacteria in synovial aspirate [9] before the diagnosis of Poncet’s can be established, but osteoarticular TB rarely involves more than one joint [10, 11] and responds rather slowly to ATT [12].

It largely remains unclear why sterile reactive polyarthritis complicates visceral TB. A vigorous immune response to mycobacteria within joints has been postulated, where mycobacterial antigen-induced activation of T cells then leads to their cross-reactivity with cartilage proteoglycans [1]. This hypersensitivity to mycobacterial antigens may be associated with HLA DR3 and/or HLA DR4 haplotypes [4]. It is interesting to note that anti-cyclic citrullinated peptide (anti-CCP) has high sensitivity and specificity for RA but now its role in active tuberculous arthritis has been reported as well [13]. Anti-CCP is no more specific to RA and frequently seen in patients with active TB [14]. This may be misleading as seen in one of our patients (case 2) who started on Salazopyrin (sulfasalazine) and methotrexate due to his positive RA factor and anti-CCP. TB arthritis can be confused easily with RA due to the same radiologic features including periarticular osteopenia and marginal erosions [15].

Although the diagnosis remains challenging for most clinicians, awareness of this entity is crucial and it needs to be dealt with at a public health care level. For developed countries, where TB has returned to center stage with the emergence of HIV, clinicians are likely to encounter more cases [16, 17]. Thereby it is imperative to recognize the ever-expanding spectrum of tuberculous manifestations.

## Conclusions

Given the debilitating nature of the illness and the ease of cure, tuberculous etiology deserves strong clinical suspicion in the differential diagnosis of polyarthritis of obscure cause or unusual presentation. In such cases, ruling out TB should take precedence over the extensive diagnostic workup required for autoimmune etiologies of similar presentation. For endemic regions, widespread knowledge may itself enhance the identification of otherwise neglected cases that may be contributing to the high mortality associated with untreated TB.
